# Population structure and phylogeography of three closely related tree peonies

**DOI:** 10.1002/ece3.10073

**Published:** 2023-06-01

**Authors:** Guangli Liu, Ge Xue, Tingting Zhao, Yang Li, Liangliang Yue, Huixing Song, Qinglin Liu

**Affiliations:** ^1^ College of Landscape Architecture Sichuan Agricultural University Chengdu China; ^2^ National Plateau Wetlands Research Center, College of Wetlands Southwest Forestry University Kunming China

**Keywords:** cross‐breeding, ecological niche modeling, lineage confluence, phylogeography, population dynamics, tree peony

## Abstract

*Paeonia decomposita*, *Paeonia rotundiloba*, and *Paeonia rockii* are three closely related species of Sect. *Moutan* is distributed in the montane area of the Eastern Hengduan Mountain region. Understanding the population history of these three tree peony species could contribute to unraveling the evolutionary patterns of undergrowth species in this hotspot area. We used one nuclear DNA marker (internal transcribed spacer region, ITS) and two chloroplast DNA markers (*matK*, *ycf1*) to reconstruct the phylogeographic pattern of the populations. In total, 228 individuals from 17 populations of the three species were analyzed in this study. Three nuclear clades (Clade I – Clade III) and four maternal clades (Clade A – Clade D) were reconstructed. Molecular dating suggested that young lineages diverged during the late Pliocene and early Pleistocene, younger than the uplift of the Hengduan Mountains but older than the last glacial maximum (LGM). Significant population and phylogeographic structures were detected at both markers. Furthermore, the populations of these tree peonies were overall at equilibrium during the climatic oscillations of the Pleistocene. The simulated palaeoranges of the three species during the LGM period mostly overlapped, which could have led to cross‐breeding events. We propose an evolutionary scenario in which mountain orogenesis around the Hengduan Mountain area triggered parapatric isolation between maternal lineages of tree peonies. Subsequent climatic fluctuations drove migration and range recontact of these populations along the valleys. This detailed evolutionary history provides new insights into the phylogeographic pattern of species from mountain‐valley systems.

## Graphical abstract

Location of populations sampled (a); Geographic distribution of the chloroplast (cp) DNA haplotypes (population codes and chlorotype node refer to Table S1), the solid black lines represent three groups according to SAMOVA analysis, and the dotted lines display six groups (b); Maximum parsimony network based on chlorotypes (c). The size of the pie is a positive correlation with the frequency of each haplotype and black dots represent haplotypes that are missing, extinct, or not observed.

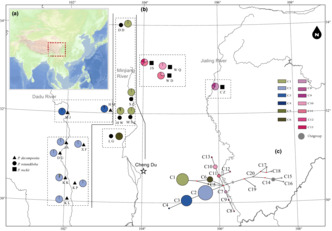

## 1 INTRODUCTION

The East Asian flora is one of the most diverse temperate flora in the world (López‐Pujol et al., 2011; Lu et al., 2018; Qi et al., 2012; Qiu et al., 2011; Wang et al., 2015). Flora from montane areas contributes to more than half of the plant diversity in East Asia, especially the mountain systems in middle to western China, such as the Qinling and Hengduan Mountains. In situ, diversification may have played a vital role in shaping the plant diversity of Hengduan forests, with geological history and climatic oscillations influencing the current distributions and genetic structures of many plant species (Favre et al., 2015; Perrigo et al., 2020; Xiang et al., 2017; Yang et al., 2022; Zhang et al., 2018). The Hengduan Mountains were thought to have experienced a series of topographical deformations from the late Oligocene (Ding et al., 2020; Su et al., 2019). Orogenic activities in mountain ranges increase topographic heterogeneity, create dispersal barriers or corridors, form novel habitat types, promote speciation, and influence phylogeographic patterns of species (Liu, Moller, et al., 2013; Yin et al., 2021). In addition, climate oscillations usually induce range contractions or expansions from refugial areas of many plants, with populations moving latitudinally along valleys (Tank & Sang, 2001; Vitt et al., 2010) and altitudinally on mountain slopes (Wang et al., 2010; Zhang et al., 2018). The glacial–interglacial cycles of the Quaternary repeatedly altered organismal distributions and triggered unstable population dynamics (Matias et al., 2017). Lineages of plant species may have opportunities to mix or to split due to isolation.

Lineage fusion and splitting were probably caused by orogeny and climate change. Lineage fusion is a special case in which two or more populations of parental lineages that were not reproductively isolated collapse into a single lineage after they meet, which has an important impact on present‐day distributions of genetic diversity by reshuffling variation (Garrick et al., 2019, 2020; Kearns et al., 2018). Lineage fusion events can be realized by horizontal gene transfer or hybridization (Niehus et al., 2015; Yue, Hu, et al., 2012). Mountain‐valley systems, a typical landscape of the Qinling–Hengduan Mountain region, are usually characterized by high mountains and deep valleys, and diverse local environments (Ding et al., 2020; Wang et al., 2022). If the orientation and morphology of mountain valleys are suitable, valleys and river drainages could serve as corridors for organisms around this area during glacial–interglacial cycles, promoting the occurrence of hybridization events and shaping the population structure of the plants here (Chen et al., 2018; Du et al., 2022; Yue, Chen, et al., 2012; Yue & Sun, 2014; Zhang et al., 2011). In contrast, lineage splitting could be the most important mode of speciation when a mountain‐valley acts as a natural geographic barrier (Yang et al., 2014; Zhang, Chen, & Sun, 2021). However, researchers need more information on detailed lineage fusion and lineage splitting in mountain‐valley ecosystems (Zhang, Qian, et al., 2021).

The wild tree peony populations provided a case study for plant lineage fusion and lineage splitting in mountain‐valley ecosystems. *Paeonia decomposita* Hand.‐Mazz, *Paeonia rotundiloba* (D.Y. Hong) D.Y. Hong, and *Paeonia rockii* (S.G. Haw & Lauener) T. Hong & J.J. Li ex D.Y. Hong are three closely related tree peonies distributed in forest margins or thickets of adjacent valleys in the Qinling–Hengduan Mountains (Hong, 2021; Zhou, 2006). *P. decomposita* is mainly distributed along the Dadu River, and westernmost to Kangding, Sichuan; *P. rotundiloba* is mainly distributed along the upper reaches of the Minjiang River, and northernmost to Diebu, Gansu; and *P. rockii* is found in a relatively large area from central to western China (Thompson et al., 1994; Zhou et al., 2014). Previous studies showed that the three closely related tree peonies were morphologically different from each other (Hong, 2011b). *P. decomposita* is much more similar to *P. rotundiloba*, except for the differences in carpel number, disk coating degree, and leaflet morphology (Hong, 2011a). However, the phylogenetic relationships of the three closely related species are still controversial, as different genetic markers have emerged (Tank & Sang, 2001; Zhao et al., 2008; Zhou et al., 2014). Such discordance may indicate the effects of incomplete lineage sorting or introgressive hybridization (Vargas et al., 2017; Xu et al., 2019). Most populations of *P. decomposita*, *P. rotundiloba*, and *P. rockii* have been facing the risk of population extinction due to human disturbance, modern habitat fragmentation, and obstacles to seed reproduction (Song et al., 2012; Yuan et al., 2011). Therefore, more studies covering large numbers of populations on lineage history and population dynamics are needed to understand the population structure and speciation history and to formulate sustainable conservation strategies for these three species.

In this study, we present an analysis of the population genetic structure, phylogeography, and historical demography of *P. rockii*, *P. decomposita*, and *P. rotundiloba* using one nuclear DNA marker (internal transcribed spacer region, ITS) and two chloroplast DNA markers (*matK*, *ycf1*) and ecological niche modeling (ENM). Our aims in this study are to (i) reveal the geographic patterns of population genetic variation of the species; (ii) elucidate the evolutionary factors that might be responsible for the patterns and levels of genetic variation observed; and (iii) describe the demographic patterns of three closely related tree peonies that evolved during the LGM.

## 2 METHODS

### 2.1 Sampling strategy

A total of 228 individuals from eight natural populations of *P. decomposita*, five populations of *P. rotundiloba*, and four populations of *P. rockii* were used for sampling from 2015 to 2016. Each sample was collected at least 20 m away from the other to magnify the allele frequency difference within each population. Fresh leaves of each individual were collected in the field and immediately dried with silica gel for DNA testing (see Tables S1 and S3). Sequences of *P. delavayi*, *P. lutea*, and *P. ludlowii* from NCBI were selected as outgroups for the phylogenetic reconstructions (accession numbers AY328312.1, U27683.1, U27679.1 for nrITS; and KP089771.1, KY817591.1, KP089772.1, KP089773.1, KP089774.1, KP089775.1, KY817592.1 for cpDNA).

### 2.2 DNA extraction, amplification, cloning, and sequencing

Total DNA was extracted from 30 mg of dried leaf tissue using a LABGENE™ Plant DNA Isolation Kit for Dry Samples (LABGENE Biotechnology Co., Ltd.). A total of three DNA fragments, the ITS and two chloroplast genes *matK* and *ycf1*, were amplified and sequenced using the primer pairs ITS‐1F (5′‐GTA GGT GAA CCT GCA GAA GGA TCA‐3′), 18S‐25S‐3′R (5′‐CCA TGC TTA AAC TCA GCG GGT‐3′; Zhu et al., 2015); *matK*472F (5′‐CCC RTY CAT CTG GAA ATC TTG GTT C‐3′), *matK*1248R (5′‐GCT RTR ATA ATG AGA AAG ATT TCT GC‐3′; Yu et al., 2011); and *ycf1*F (5′‐CAT GCC GAA GTG ATG GAA AA‐3′), and *ycf1*R (5′‐TTT CGA CGA AAA TCT GAT TGT TGC GAA T‐3′; Ding et al., 2020; Wang et al., 2022). Amplicons were purified using the GENEOUT™ DNA Extraction & Clean Kit following the manufacturer's protocol (GENEOUT Biotechnology Co., Ltd.) and sequenced immediately. PCR products from individuals who had more heterozygous sites were purified and cloned using pGEM®‐T Easy Vectors following the manufacturer's instructions. For each sample, 10–15 clones were selected for sequencing. Sequencing reactions were run on an ABI Prism 3730 automated DNA sequencer (Applied Biosystems Inc.).

### 2.3 Phylogenetic analysis

Sequences were initially subjected to a BLAST search against the GenBank database (www.ncbi.nlm.nih.gov) to detect contamination and confirm the targeted markers. Sequences were aligned using MAFFT v.7 and then adjusted manually in BioEdit v.7.0.0 (Hall, 1999). We determined nrITS types using the algorithm PHASE (Stephens & Donnelly, 2003) implemented in DnaSPv.5.0 (Librado & Rozas, 2009), using 1000 iterations with 1000 generation burn‐in iterations and a thinning interval of 10 (Stephens et al., 2001). The nrITS and concatenated cpDNA haplotypes were identified with DnaSP5 (Librado & Rozas, 2009) using *P. lutea* (AY328312.1 and U27683.1) and *P. delavayi* (U27679.1) as nrITS outgroups and *P. lutea*, *P. delavayi* (KP089771.1 and KY817591.1, KP089772.1, KP089773.1, KP089774.1, and KP089775.1), and *P. ludlowii* (KY817592.1) as cpDNA outgroups to reconstruct the phylogenetic tree. Maximum likelihood (ML) trees were reconstructed using PAUP* 4.0b10 (Swofford, 2002), and Bayesian trees were reconstructed using MrBayes 3.1.6 (Ronquist et al., 2012). All characters were weighted and unordered equally, and gaps were treated as missing data. The best nucleotide substitution models for cpDNA and nrITS were GTR and GTR + G, selected using the Akaike information criterion (AIC) in MrModeltest 2.3 (Nylander, 2004). For Bayesian analyses, two independent runs were performed through 10,000,000 generations with four Markov chains.

### 2.4 Phylogeographic and population genetic data analyses

For both cpDNA (chlorotypes) and ITS, indices of haplotype diversity (*h*) and nucleotide diversity (*π*) were estimated according to Nei (1987) at the levels of populations (*h*_S_ and *π*_S_) and species (*h*_T_ and *π*_T_) using DnaSP (Librado & Rozas, 2009). Genealogical relationships among cpDNA and nDNA haplotypes identified were constructed using the program NETWORK v.5 (available at http://www.fluxus‐engineering.com/index.htm) with the MP criterion. A split phylogenetic tree based on the Neighbor‐Net algorithm (NNet; Huson & Bryant, 2006) was also reconstructed for the nrITS haplotypes using SPLITSTREE v.4.14.6. Neighbor‐Net networks can indicate any detailed potential conflicts among different haplotypes caused by complex evolutionary events such as hybridization, polyploidization, and recombination (Kilian et al., 2007; Liu et al., 2014).

SAMOVA 1.0 (Dupanloup et al., 2002) was used to define the best groups of populations (*K*) that were geographically homogeneous and maximally differentiated from each other, with the number of groups ranging from 2 to 20. The analysis was reported five times for each *K* value with 1000 independent iterations, starting from 100 random initial conditions. The configuration with the highest and most significant *F*_CT_ value was retained as the best grouping of populations.

Genetic diversity (*H*_S_) and total genetic diversity (*H*_T_) within a population, as well as genetic differentiation between populations (*G*_ST_ and *N*_ST_), were estimated using PERMUT v1.2.1 with the 1000 permutations test (http://www.pierroton.inra.fr/genetics/labo/Software/PermutCpSSR). The *G*_ST_ and *N*_ST_ were compared to determine the phylogeographic structure following Pons and Petit (1996).

Hierarchical analysis of molecular variance (AMOVA; Excoffier et al., 1992) was performed to quantify the genetic differentiation among groups, within populations, and among populations using Arelquin v.3.0 (Excoffier et al., 2007), with 1000 random permutations. Populations were partitioned by geography or species. Geographical groups were obtained from SAMOVA Correlations between the genetic distances (*F*_ST_) and geographical distances of all populations (Mantel test) were tested using the software Arlequin v.3.0 (Excoffier et al., 2007) with 10,000 permutations at 0.05 significance levels. Pairwise geographical distance matrices were constructed by GenAlEx6 (Peakall & Smouse, 2012).

### 2.5 Demographic analyses and lineage divergence time of three closely related tree peonies

Tajima's *D* (Tajima, 1989) and Fu's *F*_S_ (Fu, 1997) were calculated in ARLEQUIN to test for deviations from neutrality. In an attempt to further infer demographic processes, we also tested the null hypothesis of spatial expansion and pure demographic expansion using mismatch distribution analysis (MDA) in ARLEQUIN (Excoffier et al., 2007) at the lineage and species levels. Goodness‐of‐fit was tested with the sum of squared deviations (SSD) between observed and expected mismatch distributions and Harpending's (Relethford & Harpending, 1994) raggedness index (*HRag*) using 1000 parametric bootstrap replicates for each model. Where the sudden expansion model was not rejected, the formula *T* = τ/2u (Rogers & Harpending, 1992) was used to estimate the age of expansion (*t*), where *u* = *μkg*; *μ* is the neutral mutation rate of the sequence in substitutions per site per year (s/s/y), *k* is the average sequence length (here, ITS, 636 bp; cpDNA, 1315 bp) and *g* is the generation time in years (Wang, 2010). Considering that there is no fossil record of *Paeonia*, a constant cpDNA substitution rate range for most angiosperm species was adopted (from 1.0 × 10^−9^ s/s/y to 3.0 × 10^−9^ s/s/y; Wolfe et al., 1987). For ITS, we adopted a nucleotide substitution rate ranging from 3.94 × 10^−9^ to 6.06 × 10^−9^ (Richardson et al., 2001).

To estimate the change in demographic growth over the history of the samples, we carried out two Bayesian skyline plot analyses (BSPs), implemented in BEAST v1.8.2 (http://beast.community/). The analyses were run using an uncorrelated relaxed molecular clock under Bayesian skyline models for 10^8^ iterations with burn‐in periods of 25%. The substitution models were set to GTR + I + G for nrITS and HKY + I for cpDNA. Genealogies and model parameters were sampled every 10,000 iterations. Chain convergence was inspected in Tracer v.1.5 software, and the results with an effective sample size (ESS) for each parameter >200 were accepted. The BSP was visualized in the program Tracer v.1.5, which summarizes the demographic history over time.

BEAST v.1.8.2 was also used to estimate divergence times since the most recent common ancestor of all the lineages. The program BEAUti (Drummond et al., 2012) was used to set criteria for the analysis, in which we applied the GTR + I + G substitution model for nrITS and the GTR + I substitution model for cpDNA, according to MrModeltest 2.3 (Nylander, 2004). Other nondefault settings were as follows: an uncorrelated relaxed molecular clock model (Drummond et al., 2002) was selected; the Yule Process was specified as the tree prior; and the prior parameters of “ucld.mean” were set to “uniform” distributions. Posterior distributions of parameters were obtained using MCMC analysis for 10^7^ generations with 25% burn‐in. The log file was then checked for convergence of the chains using Tracer 1.5. A maximum clade credibility (MCC) tree with the maximum sum of posterior probabilities on its internal nodes (Drummond et al., 2007) was adopted to summarize the samples from posterior distributions using TreeAnnotator v.1.8.2 (Drummond & Rambaut, 2007), in which the posterior probability limit was set to 0.5 summarizing mean node heights. The MCC tree was visualized and edited using FigTree v.1.4.3, and the means and 95% higher posterior densities (HPDs) of the tree were obtained from it. The 95% HPD represents the shortest interval that contains 95% of the sampled values from the posterior (Drummond et al., 2006).

### 2.6 Ecological niche modeling

To examine niche divergence between the targeted species, we used the maximum entropy model and machine‐learning algorithm implemented in MAXENT v.3.3.3k (Phillips et al., 2006; Phillips & Dudík, 2008) to predict ecological niche models based on presence‐only data obtained from our field observations, herbarium records in the Chinese Virtual Herbarium (http://www.cvh.ac.cn/) and the literature (Yuan et al., 2012) for all three species (Table S6). Current values for 19 bioclimatic variables together with the altitude data package, which was at a resolution of 2.5 (arc‐minutes), were downloaded from the WorldClim dataset set (Hijmans et al., 2005) as environmental layers. All 228 individuals were used for ENM analysis, and the number of individuals of each species was above 50. We calculated pairwise Pearson's correlation coefficients for current climate and altitude data across distributions of all tree peonies performed in ENMtools version 1.3 (Warren et al., 2010). Any factor that had a correlation coefficient greater than 0.85 with two or more other factors was excluded to increase the accuracy of the ENM prediction. Default parameters for MAXENT were adopted, and 80% of the species records were used for training and 20% for testing the model. To statistically evaluate model performance, we used the area under the “receiver operating characteristic (ROC) curve” (AUC; Fawcett, 2006), a threshold‐independent measure of model performance compared to null expectations. A model with an AUC approximating 1 indicated perfect predictive ability. We used DIVA‐GIS v.7.5 (http://www.worldclim.org/; Hijmans & Spooner, 2001) to draw the range of suitable distributions.

## 3 RESULTS

**FIGURE 1:**
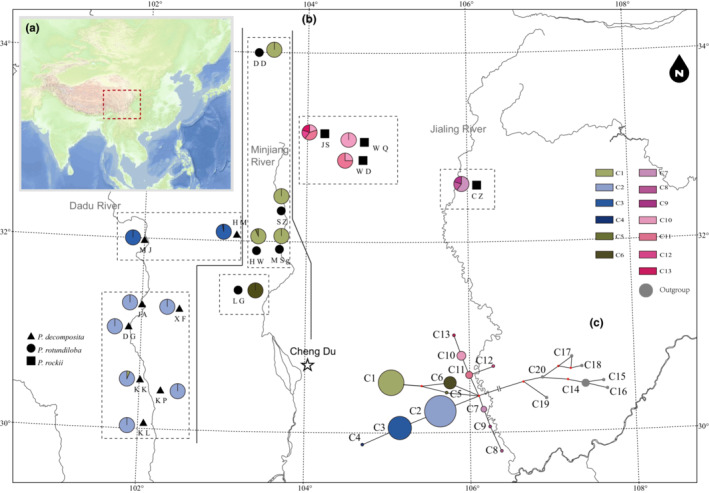
Locations of the populations sampled (a). Geographic distribution of the chloroplast (cp) DNA haplotypes (population codes and chlorotype nodes are referenced in Table S1); the solid black lines represent three groups according to SAMOVA, and the dotted lines display six groups (b). Maximum parsimony network based on chlorotypes (c). The size of the pie is positively correlated with the frequency of each haplotype, and black dots represent haplotypes that are missing, extinct, or not observed.

**FIGURE 2:**
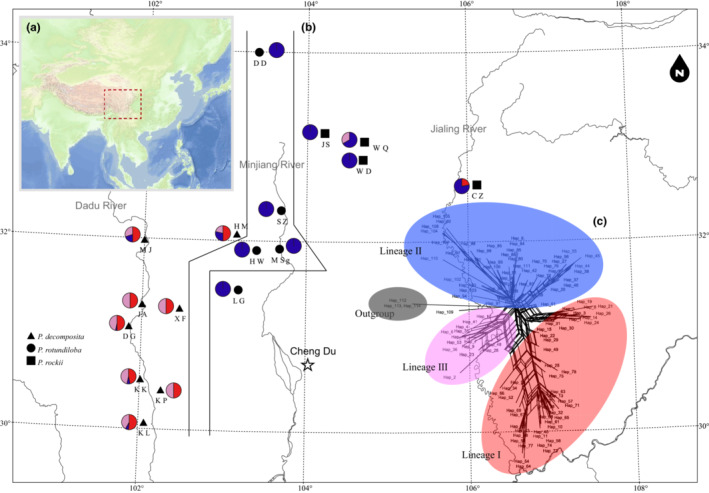
Locations of the populations sampled (a). Geographic distribution of the nrITS haplotypes (population codes and haplotype codes are referenced in Table 1 and Table S1) (b). Network based on the Neighbor‐Net (NNet) algorithm of nrITS haplotypes (c).

In this study, 221 individuals were successfully sequenced for cpDNA, and 228 individuals were sequenced for nrITS (Tables S1 and S3). The total length of concatenated chloroplast DNA alignment was 1315 bp, containing 8 nucleotide substitutions and 4 indels (insertion/deletion). The nrITS alignment length was 636 bp, revealing 58 nucleotide substitutions and three indels (1 bp). Thirteen cpDNA haplotypes (‘chlorotypes’, C1–C13; Table S1, Figure 1b) and 111 ITS haplotypes (‘ribotypes’, Hap1‐Hap111; Table S2, Figure 2b) were identified in this study.

### 3.1 Phylogenetic analyses and estimations of divergence times

**FIGURE 3:**
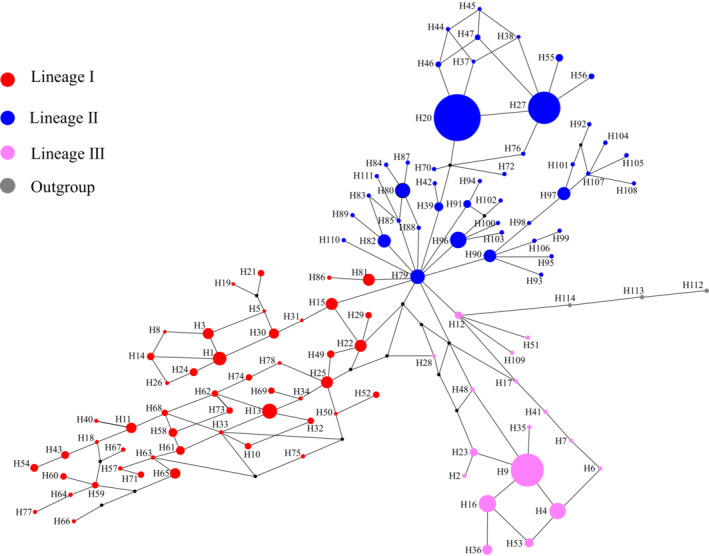
Maximum parsimony network based on nrITS haplotypes. The size of the pie is positively correlated with the frequency of each haplotype, and black dots represent haplotypes that are missing, extinct, or not observed.

**FIGURE 4:**
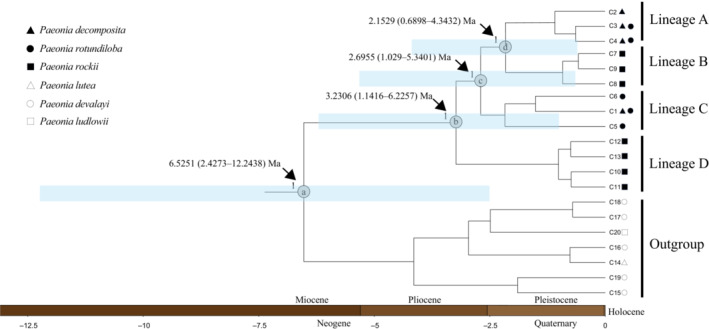
BEAST‐derived chronograms based on cpDNA haplotypes. BEAST‐derived topology is similar to Bayesian/ML/MP analyses. C1–C13 represent the ingroups; details are referenced in Table 1. C14–C20 represent the outgroups; C14, *P. lutea*; C15–C19, *P. devalayi*; C20, *P. ludlowii*.

**FIGURE 5:**
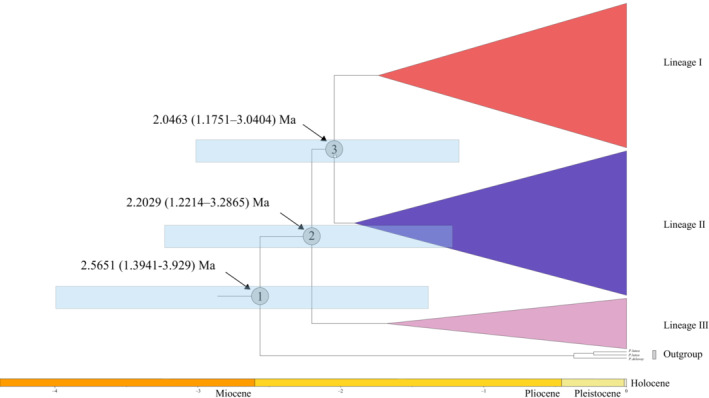
BEAST‐derived chronograms based on nrITS haplotypes. BEAST‐derived topology is similar to Bayesian/ML/MP analyses. Node values indicate mean divergence dates and 95% highest posterior density (HPD).

Four maternal clades were reconstructed (Clade A–D, Figure 4) based on chlorotype data, in which the paraphyly of *P. decomposita* and *P. rotundiloba* was confirmed in this analysis using the neighbor‐joining (NJ), Bayesian, and maximum likelihood methods. Clade C contained haplotypes from *P. decomposita* and *P. rotundiloba. P. rockii* was polyphyletic and included haplotypes C7–C9 (extracted from populations JS, WD, WQ) and C10–C13 (extracted from population CZ), corresponding to Clades B and D respectively (for details, see Table S1 and Figure 4). Four clades were connected by one median vector in the phylogenetic network (Figure 1c). For the nrITS database, the NJ tree, MrBayes tree, ML tree, and Splitstree indicated minor differences. Three main clades were recovered (Clades I–III). While Clades I and III harbored haplotypes extracted from both *P. decomposita* and *P. rockii*, Clade II contained haplotypes from all three tree peonies (for details, see Table 2, Figures 2c and 5). The phylogenetic network indicated a star‐like evolutionary pattern dominating the three clades (Figure 3).

The divergence times of the lineages ranged from the late Miocene to the Pleistocene (Figures 4 and 5). Based on the cpDNA chronogram (Figure 4), we estimated the crown time (node a) of four lineages and outgroups (c. 6.5251 Ma; 95% HPD: 2.4273–12.2438 Ma), suggesting a late Miocene to Pliocene split from the outgroups (posterior probability, PP = 1). The crown time (node b) of lineage D and the other three lineages was estimated to be c. 3.2306 Ma (95% HPD: 1.1416–6.2257 Ma), indicating a Pliocene split between lineage D and the others. The crown ages (node c) between lineage C and lineages A and B were estimated to be c. 2.6955 Ma (95% HPD: 1.029–5.3401 Ma), while the crown ages (node d) of lineages A and B were c. 2.1529 Ma (95% HPD: 0.6898–4.3432 Ma), with the lineage origin prior to the Quaternary and far earlier than the LGM. For the cpDNA chronogram, BEAST adopted substitution rates ranging from 0.3 × 10^−9^ to 1.0 × 10^−9^ substitutions per site per year (s/s/y) (Su et al., 2015). For the nrITS chronogram, the substitution rate was defined from 3.94 × 10^−9^ to 6.06 × 10^−9^ substitutions per site per year (s/s/y) (Richardson et al., 2001). We estimated the crown age of three lineages and outgroups as c. 2.5651 Ma (95% HPD: 1.3941–3.929 Ma; node 1), which fell into the late Pliocene to the early Pleistocene. The split of lineage III was estimated to be c. 2.2029 Ma (95% HPD: 1.2214–3.2865 Ma; node 2), and the lineage split between lineages I and II was dated to c. 2.0463 Ma (95% HPD: 1.1751–3.0404 Ma; node 3).

### 3.2 Phylogeographic and population structures

Significant population and phylogeographic structures were detected at both markers. The total genetic diversity (*H*_T_, 0.835 for cpDNA, 0.943 for nrITS) was higher than the average within‐population diversity (*H*_S_, 0.143 for cpDNA, 0.776 for nrITS). The *N*_ST_ was significantly higher than the *G*_ST_ values for both markers (*N*_ST_ = 0.927, *G*_ST_ = 0.829, *p* < .01 for cpDNA; *N*_ST_ = 0.365, *G*_ST_ = 0.178, *p* < .01 for nrITS), suggesting a strong phylogeographic structure within these three species. For the spatial analysis of molecular variance (SAMOVA), the gradients of distribution of *F*_CT_ values were both significantly decreased when *K* = 3 (*F*_CT_ = 0.83436, *p* < .01 for cpDNA; *F*_CT_ = 0.38275, *p* < .01 for nrITS; Figure S1). The *F*_CT_ values reached a plateau (*F*_CT_ = 0.95864, *p* < .01 for cpDNA; *F*_CT_ = 0.39078, *p* < .01 for nrITS; Figure S1) when the sampled populations were defined into six groups (*K* = 6). Six groups can be concluded as a subdivision of the three groups' design (Figures 1b and 2b).

When *K* = 3, the West group of the two markers contained all the *P. decomposita* populations. This group was distributed mainly along the valleys of the Dadu River of northwestern Sichuan Province, except for the HM population. For the cpDNA marker, the West group contained four haplotypes (C1–C4), three of which were endemic (C2, C3, and C4). For nrITS, the West group contained 71 haplotypes (see Table S2 for details), which could be clustered into three lineages (red 49.64%, blue 10.95%, and purple 39.42%). The Central groups of the two markers were both located mainly along the valleys of the Min‐Jiang River in northwestern Sichuan Province. For the cpDNA, the Central group consisted of four populations of *P. rotundiloba* (DD, SZ, HW, and MSg) and contained two haplotypes (C1 and C5), only one of which was endemic for *P. rotundiloba* (C5). For nrITS, the Central group of nrITS included all five *P. rotundiloba* populations, and 11 haplotypes (H20, H27, H37–H39, H44–H47, H55–H56) clustered into the blue lineage, eight of which (H37–H38, H44–H47, H55–H56) were endemic. The East group of cpDNA contained one *P. rotundiloba* population (LG) and all four *P. rockii* populations, corresponding to eight haplotypes (C5, C7–C13), all of which were endemic. In contrast, the East group of nrITS contained all four *P. rockii* populations and 33 haplotypes (H79–H111), all of which were endemic and clustered into three lineages (red 8.33%, blue 90.63%, and purple 1.04%). Each population contributed quite differently to total haplotype diversity. For details of the haplotype geographic distribution, see Figures 1b and 2b and Tables S1 and S2.

AMOVA revealed that the differences among populations (for total populations) explained 91.7% of the total cpDNA variation. However, variation within populations accounted for most (83.68%) of the variation in nrITS (Figures S2 and S3). For the three groups identified by SAMOVA, AMOVA showed that a large proportion (52.86%) of the cpDNA variation was partitioned among groups, suggesting a significant regional population substructure. In contrast, only a small proportion (16.11%) of the nrITS variation was distributed among groups (Tables 1 and 2).

**TABLE 1:** The analysis of molecular variance (AMOVA) for cpDNA data.

Source of variation	df	Sum of squares	Variance components	Percentage of variation	Fixation indices
(i) Total populations					
Among populations	16	75.725	0.36740 V_a_	91.70	*F*_ST_ = 0.91697**
Within populations	204	6.786	0.03327 V_b_	8.30	
Total	220	82.511	0.40067		
(ii) Three groups					
Among groups	2	39.203	0.26486 V_a_	52.86	*F*_ST_ = 0.93361**
Among populations within groups	14	36.522	0.20292 V_b_	40.50	*F*_SC_ = 0.85915**
Within populations	204	6.786	0.03327 V_c_	6.64	*F*_CT_ = 0.52861**
(ii) Six groups					
Among groups	5	73.877	0.44717 V_a_	91.06	*F*_ST_ = 0.93225**
Among populations within groups	11	1.848	0.01061 V_b_	2.16	*F*_SC_ = 0.24190**
Within populations	204	6.786	0.03327 V_c_	6.77	*F*_CT_ = 0.91064**
(ii) Three species					
Among species	2	32.559	0.22438 V_a_	45.61	*F*_ST_ = 0.93238**
Among populations within species	14	43.166	0.23431 V_b_	47.63	*F*_SC_ = 0.87567**
Within populations	204	6.786	0.03327 V_c_	6.76	*F*_CT_ = 0.45610**

**p < .01.

**TABLE 2:** The analysis of molecular variance (AMOVA) for nrITS data.

Source of variation	df	Sum of squares	Variance components	Percentage of variation	Fixation indices
(i) Total populations					
Among populations	16	43.785	0.07660 V_a_	16.32	*F*_ST_ = 0.16317**
Within populations	509	199.968	0.39286 V_b_	83.68	
Total	525	243.753	0.46947		
(ii) Three groups					
Among groups	2	28.142	0.08010 V_a_	16.11	*F*_ST_ = 0.2099**
Among populations within groups	14	15.643	0.02433 V_b_	4.89	*F*_SC_ = 0.05831**
Within populations	509	199.968	0.39286 V_c_	79.00	*F*_CT_ = 0.16108**

**p < .01.

### 3.3 Demographic analyses

The null hypotheses of population expansion were rejected. No significant Tajima's *D* values (cpDNA: *D* = 2.28350, *p* > .05, Table S4; nrITS: *D* = −0.729, *p* > .05, Table S5) or *F*_S_ values (cpDNA: *F*_S_ = 0.189, *p >* .05, Table S4; nrITS: *F*_S_ = −24.52, *p* < .01, Table S5) were found, thereby not supporting a model of historical demographic expansion. The mismatch distribution analyses of the two types of markers (nrITS and cpDNA) showed multimodal patterns suggesting the equilibrium status of the populations (Figures S6 and S7). Statistical comparisons between these observed and simulated distributions under a sudden expansion model for two markers significantly rejected the expansion model (*p* > .05 based on SSD and HRag, Tables S4 and S5). The BSP analysis of cpDNA revealed that the effective population sizes were stable during the middle Pleistocene and showed a slight decrease during the late Pleistocene (Figure S4), while the effective population sizes based on nrITS were shown to be slightly increased during the middle Pleistocene, albeit along with a significant increase approximately 120,000 years ago (Figure S5).

### 3.4 Present and past (LGM) distribution

**FIGURE 6:**
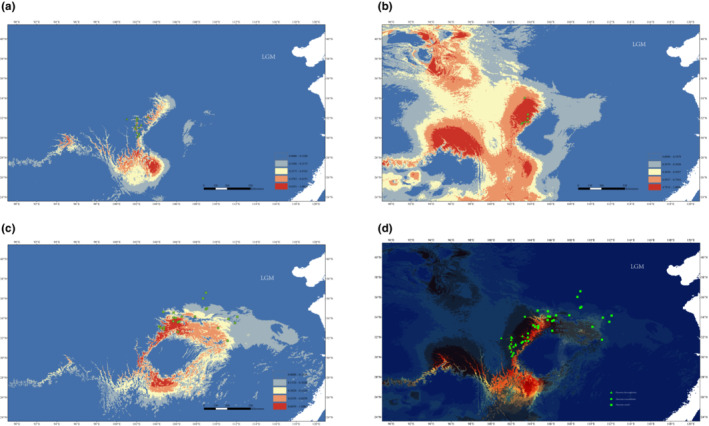
Predicted distribution ranges of *P. decomposita* (up triangle, a), *P. rotundiloba* (circle, b), and *P. rockii* (square, c) in the LGM and the overlapping distribution ranges of the three species in the LGM (d).

The AUC values for the current potential distributions of *P. decomposita*, *P. rotundiloba*, and *P. rockii* indicated good predictive model performance (0.990, 0.995, and 0.992, respectively). The current distributional predictions covered the species' extant distribution. However, there were also some predicted areas where the species are not found at present. In contrast, distributional predictions for the LGM (c. 21,000 year BP) were different from the present, revealing a general southwards range shift. Furthermore, the simulated distribution areas of the three tree peonies at the LGM partially overlapped (Figure 6).

## 4 DISCUSSION

### 4.1 Genetic diversity and phylogeographic pattern

The three tree peonies are characterized by high genetic diversity at the overall population level in both cpDNA and nrITS markers. In this study, the *h*_T_ values of cpDNA and nrITS were as high as 0.835 and 0.943, respectively, and were significantly higher than those reviewed by Qiu et al. (2011). The three tree peonies mainly propagate by seeds (Cheng et al., 1997), which are normally dispersed by either gravity or rats (Hong, 2011b). Individuals always start to flower 4–6 years after germination, and it is generally assumed that the generation time of *Paeonia* species is 10 years (Sang et al., 1997; Xu et al., 2019). The long generation time of tree peonies may have acted as an intrinsic buffer against the loss of genetic diversity because individuals of different generations could share the same habitat (Hailer et al., 2006). Individual tree peonies could live for more than 30 years according to our long‐term field observations by counting bud scale scars. As a result, there could be more than 20 generations of individuals that could contribute to the gene pool and accumulate the overall genetic diversity for the populations.

Furthermore, tree peonies can produce an average of more than 100 seeds per individual per year. The effective population size could be relatively high; for example, dozens to over a thousand individuals per hectare were found in our field work. The high effective population size could reduce the effect of genetic drift, which could accumulate haplotype diversity in the maternal system (Chen et al., 2017; Montarry et al., 2019). For nrITS markers, tree peonies are pollinated by insects such as bees, and pollen flow via insects can make tree peonies exhibit a more uniform distribution of diversity even in sparse *Cupressus chengiana* forests, young secondary deciduous broad‐leaved forests (Wang, 2020; Xu et al., 2019; Yuan et al., 2011). In addition, hybridization, either cross‐species or intraspecific hybridization, is increasingly recognized as a generator of diversity (Lamichhaney et al., 2018; Li et al., 2020). Our results were consistent with several previous studies in the Hengduan Mountains, such as those studying *Ligularia tongolensis* (Wang et al., 2011), *Terminalia franchetii* (Zhang et al., 2011), *Cyananthus delavayi* (Li et al., 2012), *Lespedeza buergeri* (Jin et al., 2016), and *Forsythia suspense* (Li et al., 2011).

The three species of tree peonies showed significant genetic differentiation and phylogeographic structure within distinct topographical differences among the western Qionglai Mountains, the Songpan Plateau, and the eastern Min Mountains. However, the levels of genetic differentiation and genetic structure among populations were likely to be higher for the cpDNA markers than for nrITS (obtained via comparisons of *G*_ST_ and *N*_ST_ and AMOVA). Mountain barriers could shape the phylogeographic patterns and population structures of these tree species. The Min Mountains (4000 m a.s.l.) serve as a geographical barrier, as steep mountains and thick forest vegetation prevent seed and pollen dispersal from crossing, to realize gene transfer between tree peony recipients and potential donors. The role of landscape features in affecting *genetic differentiation* has been documented in the *Paeonia* subsect. *Delavayanae* (Zhang et al., 2018), *Cyananthus delavayi* (Li et al., 2012), *Quercus aquifolioides* (Du et al., 2017), and *Buddleja crispa* (Yue, Chen, et al., 2012).

Different levels of genetic differentiation and genetic structure among populations between cpDNA and nrITS could be attributed to the characteristics of their different inheritance systems. The cpDNA is maternally inherited with low recombination and mutation rates and is free of the types of phenomena that lead to violation of crossbreeding (Mark et al., 2007; Schaal et al., 1998). In contrast, the nuclear rDNA ITS is parentally inherited with relatively higher recombination and substitution rates (Mark et al., 2007). Moreover, insect‐triggered pollen flows carrying genes, such as ITSs, are always stronger and farther from the original individuals than the seed flows, which may increase the gene introgression between the populations, lead to the unification processes of alleles and form a lower phylogeographic structure across the range.

### 4.2 Lineage sorting and fusion of the three *Paeonia* species

Complete lineage sorting was observed from the chloroplast segments. (Figures 4 and 1c). The splits of the main lineages were dated from the late Pliocene to the early Pleistocene (c. 3.23 Ma to c. 2.15 Ma) for the cpDNA markers (Figure 4), both of which were prior to the Quaternary glaciation age. Around this time, the Qinghai‐Tibet Plateau (QTP) was subjected to the Qingzang Movement, followed by the onset of the uplift of the Hengduan Mountains, forming a mountain–valley landscape in this and adjacent areas (Clark et al., 2005; Wang et al., 2012; Xing & Ree, 2017) and inducing isolation and allopatric speciation events. Close correlations between lineage splitting and geologic changes have also been observed in other plants, e.g., *Tetrastigma hemsleyanum* (Wang et al., 2015), *Taxus wallichiana* (Liu, Moller, et al., 2013), *Picea purpurea* (Sun et al., 2014), *Hippophae tibetana* (Wang et al., 2010), and *Picea likiangensis* (Li et al., 2013). In this study, the uplift of the Hengduan Mountains could also lead to the formation of new lineages of the three tree peonies.

From the nuclear genome data, lineage fusion was detected among the three tree peonies (Figures 5, 2c and 3). There are several reasons for lineage fusion (Yue, Hu, et al., 2012), and hybridization events could more reasonably explain the fusion of the lineages in this study. Due to the concerted evolution of intra‐ and interchromosomal loci in the long‐term process of evolution, multiple copies of the nuclear ribosomal ITS (nrITS) within a genome tend to highly homogenize the variation among nrITS repeats, as the nrITS can be found inside individuals of one species (Vollmer & Palumbi, 2004). Lineages of nrITS were split prior to the early Pleistocene (Figure 5), which coincides with the intense uplift of the QTP. From the early Pleistocene to the present, the rate of concerted evolution is sufficient to make the ITS remain a single homogeneous copy (Baldwin et al., 1995; Leitch et al., 2004). Older lineages occurring in younger individuals and populations indicate hybridization events, with perhaps more than one event, leading to the lineage fusion pattern of the three tree peonies.

### 4.3 Population dynamics during the Quaternary leading to lineage fusion

Lineage fusion could be realized by population expansions or migration induced by climate and geologic changes. Demographic or range expansions may lead to short‐distance pollen flow exchange, as well as massive gene introgression of different species (Excoffier et al., 2009; Niehus et al., 2015). We performed demographic and ecological niche modeling analyses to determine lineage fusion. However, multiple lines of evidence from the neutrality test, mismatch distribution analysis, and BSP analysis presented here suggested that range expansions or contractions did not occur during the LGM (Figures S4 and S5).

However, the Quaternary climatic fluctuation and plateau uplift have largely shaped the current patterns of species diversity (Xing & Ree, 2017; Yu et al., 2019). Climate oscillations impel latitudinal/altitudinal shifts in species distribution ranges, which may increase the chance of hybridization between species at their boundaries (Garroway et al., 2010; Mimura et al., 2014). Because of the extremely cold climate during the LGM, the three closely related peonies migrated to the lower plate through the valleys between mountains (Figure 6). Therefore, the ranges of these three species probably overlapped at least in part (in the Chengdu Plain according to the current geographical division), which would have provided an opportunity for short‐distance pollen dispersal and further hybridization (Schweiger et al., 2008).

Insect pollinators are always extremely efficient. Pollen flows carried by insects could unify the ITS copies among the three species as soon as possible once the metapopulation around the Chengdu Plain area occurred. We performed demographic and ecological niche modeling analyses to determine lineage fusion. Ecological niche modeling showed that the simulated core distribution areas during the LGM were not far from the present distribution areas for each tree peony (Figure 6). Similar results have been observed in Europe, North America, and the middle and eastern Tibetan Plateau, revealing that long‐distance migration from old core distribution areas to new ones can follow climatic oscillations (Clayton et al., 2009; Givnish et al., 2016; Liu, Ickert‐Bond, et al., 2013; Nakamura et al., 2012).

### 4.4 Conservation strategies for three closely related species

Due to the frequent occurrence of geological disasters in the distribution of peony in Southwest China, many wild resources have been destroyed. The pollination period coincides with the rainy season, which reduces insect pollination efficiency. Moreover, with the promotion of peony oil in recent years, the picking of wild germplasm resources has become more frequent, which is not conducive to the natural renewal of wild populations and results in smaller population size. Small‐scale populations do not exchange genes with the outside world and are more likely to face the risk of genetic drift. In the long run, the plant resources of the peony group are bound to face the danger of extinction. In addition, this study shows that the populations of the three related species of the tree peony group contain abundant haplotypes, and the haplotypes contained in each species are highly endemic. Therefore, we believe that existing populations can be protected in situ as protection units, and these populations can be classified into nature reserves to reduce the effects of human disturbance. Second, we encourage research on the mechanism of peony endangerment. Finally, relocation protection can be carried out. Our field investigations found that peony regeneration is better in cases of less human disturbance, which can provide better conditions for peony reproduction through introduction and conservation.

## 5 CONCLUSIONS

Our study of *P. rockii*, *P. decomposita*, and *P. rotundiloba*, integrating molecular phylogeography, and ecological niche modeling, strongly suggests that the observed patterns of genetic variation and evolutionary history are best explained by a combination of late Miocene and late Pliocene geological activity and late Quaternary climatic fluctuation.

Furthermore, valleys among mountains acted as corridors, playing important roles in lineage confluence and species migration. The glacial periods could have triggered the populations to migrate southwards but could have not killed all the populations in the deep valleys due to niche diversity. Populations in the valley and the Chendu Plain could once have formed a unified meta‐population in the shape of a character “E”, across which pollens carrying the ITSs could have been introduced into the valley populations through hybridization events.

The results of this study will increase our comprehensive understanding of the origin, evolution, phylogenetic relationship, and distribution of sect. *Moutan*. According to our results, we propose that these three tree peonies are not fully fledged sister species but instead are geographical races, semispecies, or subspecies that are undergoing an allopatric phase of divergence. However, due to the evaluation of limited samples and only a few molecular markers, these results may need validation through additional research. In a future study, multilocus approaches using many genes, or even a whole‐genome sequencing approach, should be applied to resolve the species status of these three closely related tree peonies.

## AUTHOR CONTRIBUTIONS

**Guangli Liu:** Conceptualization (lead); data curation (lead); formal analysis (lead); methodology (lead); software (lead); writing – original draft (lead); writing – review and editing (lead). **Ge Xue:** Conceptualization (lead); data curation (lead); formal analysis (lead); funding acquisition (lead); investigation (lead); methodology (lead); project administration (lead); resources (lead); visualization (lead). **Tingting Zhao:** Data curation (supporting); formal analysis (supporting); software (supporting); visualization (supporting); writing – original draft (supporting); writing – review and editing (supporting). **Yang Li:** Data curation (supporting); investigation (supporting); writing – original draft (supporting); writing – review and editing (supporting). **Liangliang Yue:** Data curation (lead); formal analysis (lead); investigation (lead); methodology (lead); software (lead); validation (lead); visualization (lead); writing – original draft (lead); writing – review and editing (lead). **Huixing Song:** Conceptualization (supporting); methodology (supporting). **Qinglin Liu:** Conceptualization (supporting); methodology (supporting).

## FUNDING INFORMATION

None.

## CONFLICT OF INTEREST STATEMENT

The authors declare no conflicts of interest.

## Supporting information


Appendix S1:

## Data Availability

The ITS sequences of per individual of have been deposited in GenBank *P. rotundiloba* and *P. decomposita* (accession numbers OM570335–OM570549), *P. rockii* (accession numbers OM562281–OM562376). The *ycf1* sequences of per individual have been deposited in GenBank (accession numbers Om993806–om994027). The *matK* sequences of per individual have been deposited in GenBank (accession numbers Om993584–om993805).
