# CORRIGENDUM

**DOI:** 10.1002/ece3.10237

**Published:** 2023-06-23

In the recent article by Liu et al. (2023), the authors would like to change the author sequence order as shown below:

Guangli Liu* | Ge Xue* | Tingting Zhao | Yang Li | Liangliang Yue | Huixing Song | Qinglin Liu

*These two authors shared first authorship and contributes equally.

The online version has been corrected online.

## References

[ece310237-bib-0001] Liu, G. , Xue, G. , Zhao, T. , Li, Y. , Yue, L. , Song, H. , & Liu, Q. (2023). Population structure and phylogeography of three closely related tree peonies. Ecology and Evolution, 13, e10073. 10.1002/ece3.10073 37274151 PMC10234759

