# Supplementary material for: Population structure and phylogeography of three closely related tree peonies
Source: Ecol Evol. 2023 Jun 1;13(6):e10073. doi: 10.1002/ece3.10073 (PMC10234759; doi:10.1002/ece3.10073)

Supplement materials

Table S1 Geographic locations and population sizes of tree peony species

| Code | species | Population /Locality | Latitude | Longitude | Altitude | N | cpDNA | |
| --- | --- | --- | --- | --- | --- | --- | --- | --- |
|  |  |  | (N) | (E) | (m) |  | Chlorotype nos. | Lineage |
| 1 | *Paeonia decomposita* | Lucheng,Kangding (KL) | 30°04′44″ | 102°05′13″ | 1920-2016 | 15 | C2(15) | A |
| 2 | *Paeonia decomposita* | Pengta,Kangding (KP) | 30°25′29.2″ | 102°16′40.4″ | 2236 | 14 | C2(14) | A |
| 3 | *Paeonia decomposita* | Kongyu,Kangding (KK) | 30°31′58.5″ | 102°01′36.9″ | 2212 | 15 | C1(1),C2(14) | A,C |
| 4 | *Paeonia decomposita* | Geshizha,Danba (DG) | 31°05′01″ | 101°51′49″ | 2020 | 8 | C2(8) | A |
| 5 | *Paeonia decomposita* | Anning, Jinchuan (JA) | 31°19′14.1″ | 102°00′59.2″ | 2114 | 15 | C2(15) | A |
| 6 | *Paeonia decomposita* | Jiaomuzu,Maerkang (MJ) | 31°59′56″ | 102°01′07″ | 2532 | 12 | C3(12) | A |
| 7 | *Paeonia decomposita* | Fubian,Xiaojin (XF) | 31°17′16.5″ | 102°28′34.7″ | 2843 | 14 | C2(14) | A |
| 8 | *Paeonia decomposita* | Ganbao,Lixian (LG) | 31°29′52″ | 103°11′21.1″ | 2232 | 15 | C6(15) | C |
| 9 | *Paeonia decomposita* | Mawo,Heishui (HM) | 32°5′2.6″ | 103°9′23.8″ | 2191 | 33 | C3(32),C4(1) | A |
| 10 | *Paeonia rotundiloba* | Wabo,Heishui (HW) | 31°55′1.06″ | 103°24′17.24″ | 2120 | 15 | C1(14),C5(1) | C |
| 11 | *Paeonia rotundiloba* | Zhenjiangguan,Songpan (SZ) | 32°20′29″ | 103°42′7″ | 2530 | 15 | C1(15) | C |
| 12 | *Paeonia rotundiloba* | Diangan, Diebu (DD) | 34°0′48″ | 103°23′20″ | 2355 | 16 | C1(16) | C |
| 13 | *Paeonia rotundiloba* | Shidaguan,Maoxian (MSg) | 31°56′11″ | 103°41′17.2″ | 2055 | 15 | C1(15) | C |
| 14 | *Paeonia rockii* | Shuanghe,Jiuzhaigou (JS) | 33°9′45″ | 104°14′5″ | 1751 | 5 | C10(1),C11(2),C12(1),C13(1) | D |
| 15 | *Paeonia rockii* | Danbao,Wenxian (WD) | 32°52′55″ | 104°43′ | 1622-1680 | 4 | C10(1), C11(3) | D |
| 16 | *Paeonia rockii* | Qiaotou,Wenxian (WQ) | 33°4′26″ | 104°44′ | 1600-1980 | 5 | C10(5) | D |
| 17 | *Paeonia rockii* | Zengjia,Chaotian (CZ) | 32°36′55″ | 106°8′10″ | 1233 | 5 | C7(3), C8(1), C9(1) | D |

Abbreviation: N, number of individuals

Table S2 Haplotypes and lineages within each population based on nrITS

| Population | N | nrITS Haplotype nos | Lineage |
| --- | --- | --- | --- |
| KL | 15 | H1(2),H3(1),H4(1),H9(8),H14(1),H15(1),H16(2),H20(2),H21(2),H22(2),H23(1),H24(2),H25(3),H26(1),H36(1) | Ⅰ, Ⅱ, Ⅲ |
| KP | 15 | H1(4),H9(10),H15(1),H16(3),H22(3),H23(2),H30(5),H31(1),H32(1), | Ⅰ, Ⅲ |
| KK | 15 | H4(1),H9(7),H10(1),H11(1),H12(3),H13(4),H14(1),H15(1),H16(2),H17(1),H18(1),H19(1),H20(2),H25(1),H32(1),H49(1),H50(1) | Ⅰ, Ⅱ, Ⅲ |
| DG | 14 | H1(4),H2(1),H3(5),H4(6),H5(1),H6(1),H7(1),H8(1),H9(3),H10(1),H40(1),H41(1),H42(1),H43(1) | Ⅰ, Ⅱ, Ⅲ |
| JA | 15 | H4(1),H9(7),H13(4),H14(1),H15(3),H16(2),H22(2),H25(1),H33(1),H34(1),H35(1),H36(3),H48(1),H49(2) | Ⅰ, Ⅲ |
| MJ | 15 | H9(6),H11(5),H16(2),H20(6),H43(2),H53(1),H54(3),H68(1),H69(1),H74(1),H77(1),H78(1) | Ⅰ, Ⅱ, Ⅲ |
| XF | 15 | H1(1),H4(1),H9(9),H13(4),H15(2),H16(1),H22(1),H24(1),H25(3),H28(1),H29(2),H36(1),H51(1),H52(1),H53(1) | Ⅰ, Ⅲ |
| LG | 15 | H20(15),H27(10),H55(3),H56(2) | Ⅱ |
| HM | 33 | H4(4),H9(5),H16(3),H20(8),H27(6),H39(2),H52(1),H53(2),H57(1),H58(4),H59(2),H60(2),H61(4),H62(2),H63(1),H64(1),H65(5),H66(1),H67(1),H68(1),H69(1),H70(1),H71(2),H72(1),H73(2),H74(1),H75(1),H76(1) | Ⅰ, Ⅱ, Ⅲ |
| HW | 17 | H20(20),H27(8),H44(1),H45(1),H46(2),H47(2) | Ⅱ |
| SZ | 15 | H20(27),H27(3) | Ⅱ |
| DD | 16 | H20(16),H27(12),H37(1),H38(1),H39(2) | Ⅱ |
| MSg | 15 | H20(15),H27(15) | Ⅱ |
| JS | 6 | H79(4),H90(5),H91(3),H92(1),H93(1),H94(1),H95(1),H96(14),H97(8),H98(1),H99(1),H100(1),H101(1),H102(1),H103(1),H104(1),H105(1),H106(1),H107(1),H108(1) | Ⅱ |
| WD | 1 | H79(4),H90(3),H97(1) | Ⅱ |
| WQ | 1 | H109(1),H110(1),H101(1) | Ⅱ, Ⅲ |
| CZ | 5 | H79(3),H80(10),H81(7),H82(9),H83(1),H84(1),H85(1),H86(1),H87(1),H88(1),H89(1) | Ⅰ, Ⅱ |

Table S3 PCR primers used for amplification of ITS and chloroplast gene (*mat*K, *ycf1*)

| Gene | Primers | 5’ Sequences 3’ | Reference |
| --- | --- | --- | --- |
| ITS | ITS-1F | GTA GGT GAA CCT GCA GAA GGA TCA | (Zhu, S., et al.,2015) |
|  | 18S-25S-3′R | CCA TGC TTA AAC TCA GCG GGT |  |
| *mat*K | *mat*K472F | CCC RTY CAT CTG GAA ATC TTG GTT C | (YU, J., et al.,2011) |
|  | *mat*K1248R | GCT RTR ATA ATG AGA AAG ATT TCT GC |  |
| *ycf1* | F | ACA CAT GCC GAA GTG ATG GAA AA | (Wenpan Dong, C.X.C.L., 2015) |
|  | R | TTT CGA CGA AAA TCT GAT TGT TGC GAA T |  |

Table S4 Results of neutrality tests and mismatch distribution analysis of different levels based on cpDNA data

| Population | Tau | ET (t, year) | SSD | p-value | Raggedness | p-value | Fu's FS | p-value | Tajima's D | p-value |
| --- | --- | --- | --- | --- | --- | --- | --- | --- | --- | --- |
| DD | 0.00 | 0.00 | 0.000 | 0.000 | 0.000 | 0.000 | 0.000 | N.A. | 0.000 | 1.00 |
| DG | 0.00 | 0.00 | 0.000 | 0.000 | 0.000 | 0.000 | 0.000 | N.A. | 0.000 | 1.00 |
| HM | 3.00 | 1754899.00 | 0.005 | 0.062 | 0.890 | 0.861 | -0.482 | 0.146 | -1.502 | 0.03 |
| HW | 3.21 | 1880573.00 | 0.025 | 0.093 | 0.787 | 0.698 | 0.235 | 0.289 | -1.491 | 0.05 |
| JA | 0.00 | 0.00 | 0.000 | 0.000 | 0.000 | 0.000 | 0.000 | N.A. | 0.000 | 1.00 |
| KK | 3.00 | 1754899.00 | 0.026 | 0.064 | 0.787 | 0.795 | 1.738 | 0.753 | -1.911 | 0.01 |
| KL | 0.00 | 0.00 | 0.000 | 0.000 | 0.000 | 0.000 | 0.000 | N.A. | 0.000 | 1.00 |
| KP | 0.00 | 0.00 | 0.000 | 0.000 | 0.000 | 0.000 | 0.000 | N.A. | 0.000 | 1.00 |
| LG | 0.00 | 0.00 | 0.000 | 0.000 | 0.000 | 0.000 | 0.000 | N.A. | 0.000 | 1.00 |
| MJ | 0.00 | 0.00 | 0.000 | 0.000 | 0.000 | 0.000 | 0.000 | N.A. | 0.000 | 1.00 |
| MSg | 0.00 | 0.00 | 0.000 | 0.000 | 0.000 | 0.000 | 0.000 | N.A. | 0.000 | 1.00 |
| SZ | 0.00 | 0.00 | 0.000 | 0.000 | 0.000 | 0.000 | 0.000 | N.A. | 0.000 | 1.00 |
| XF | 0.00 | 0.00 | 0.000 | 0.000 | 0.000 | 0.000 | 0.000 | N.A. | 0.000 | 1.00 |
| GZ | 1.28 | 747200.90 | 0.008 | 0.847 | 0.110 | 0.905 | -0.475 | 0.190 | 0.000 | 1.00 |
| WQ | 0.00 | 0.00 | 0.000 | 0.000 | 0.000 | 0.000 | 0.000 | N.A. | 0.000 | 1.00 |
| JS | 1.58 | 922006.40 | 0.045 | 0.425 | 0.250 | 0.444 | -1.648 | 0.046 | 0.000 | 1.00 |
| WD | 0.76 | 446721.30 | 0.022 | 0.676 | 0.250 | 0.935 | 0.172 | 0.345 | 0.000 | 1.00 |
| Group(3)1 | 0.64 | 373600.50 | 0.023 | 0.024 | 0.201 | 0.006 | 0.075 | 0.519 | -1.047 | 0.15 |
| Group(3)2 | 3.00 | 1754899.00 | 0.002 | 0.032 | 0.938 | 0.924 | -1.037 | 0.080 | -1.442 | 0.03 |
| Group(3)3 | 2.76 | 1616654.00 | 0.046 | 0.092 | 0.122 | 0.135 | -1.287 | 0.261 | 1.648 | 0.94 |
| Group(6)1 | 3.00 | 1754899.00 | 0.003 | 0.042 | 0.917 | 0.902 | -0.764 | 0.108 | -1.475 | 0.04 |
| Group(6)2 | 0.97 | 567826.80 | 0.028 | 0.210 | 0.194 | 0.188 | -0.870 | 0.127 | 0.000 | 1.00 |
| Group(6)3 | 1.28 | 747200.90 | 0.008 | 0.826 | 0.110 | 0.886 | -0.475 | 0.183 | 0.000 | 1.00 |
| Group(6)4 | 3.00 | 1754899.00 | 0.001 | 0.031 | 0.952 | 0.941 | -0.193 | 0.194 | -1.937 | 0.00 |
| Group(6)5 | 3.00 | 1754899.00 | 0.002 | 0.030 | 0.938 | 0.934 | -1.037 | 0.080 | -1.442 | 0.04 |
| Group(6)6 | 0.00 | 0.00 | 0.000 | 0.000 | 0.000 | 0.000 | 0.000 | N.A. | 0.000 | 1.00 |
| Paeonia | 5.58 | 3263012.58 | 0.042 | 0.182 | 0.074 | 0.281 | 0.189 | 0.591 | 2.284 | 0.98 |

Note：ET, Expansion time; SAMOVA analysis result see Fig.1

Table S5 Results of neutrality tests, mismatch distribution analysis and expansion time of different levels based on nrITS data

| Population | Tau | ET(t,year) | *SSD* | p-value | *Raggedness* | p-value | Fu's *F*_S_ | p-value | Tajima's *D* | p-value |
| --- | --- | --- | --- | --- | --- | --- | --- | --- | --- | --- |
| DD | 0.926 | 145792.1 | 0.009 | 0.260 | 0.117 | 0.191 | -0.955 | 0.262 | -0.336 | 0.431 |
| DG | 8.389 | NC | 0.028 | 0.171 | 0.046 | 0.229 | -2.231 | 0.191 | 1.576 | 0.957 |
| HM | 9.445 | NC | 0.005 | 0.731 | 0.007 | 0.953 | -7.820 | 0.023 | 3.268 | 1.000 |
| HW | 0.924 | 145485 | 0.001 | 0.756 | 0.074 | 0.599 | -1.930 | 0.093 | 0.405 | 0.701 |
| JA | 8.102 | NC | 0.006 | 0.890 | 0.014 | 0.949 | -2.645 | 0.156 | 1.222 | 0.920 |
| KK | 8.018 | NC | 0.007 | 0.715 | 0.022 | 0.593 | -4.435 | 0.046 | 1.800 | 0.977 |
| KL | 7.014 | NC | 0.017 | 0.159 | 0.024 | 0.534 | -3.331 | 0.084 | 1.750 | 0.976 |
| KP | 6.680 | NC | 0.029 | 0.328 | 0.042 | 0.506 | 0.279 | 0.589 | 0.665 | 0.794 |
| LG | 1.000 | 157480.3 | 0.009 | 0.227 | 0.121 | 0.214 | -0.040 | 0.473 | 0.233 | 0.644 |
| MJ | 7.363 | NC | 0.010 | 0.439 | 0.019 | 0.617 | 0.373 | 0.597 | 3.308 | 1.000 |
| MSg | 0.797 | 125492.9 | 0.026 | 0.021 | 0.269 | 0.026 | 1.709 | 0.752 | 1.635 | 0.975 |
| SZ | 2.965 | 466903.9 | 0.016 | 0.255 | 0.429 | 0.430 | 0.037 | 0.228 | -0.409 | 0.223 |
| XF | 6.900 | NC | 0.006 | 0.793 | 0.020 | 0.764 | -3.450 | 0.078 | 1.304 | 0.918 |
| CZ | 2.426 | 382012.6 | 0.021 | 0.101 | 0.070 | 0.228 | -3.787 | 0.023 | 0.130 | 0.584 |
| WQ | 7.879 | NC | 0.158 | 0.466 | 0.444 | 0.859 | 0.807 | 0.419 | 0.000 | 0.702 |
| JS | 6.783 | NC | 0.016 | 0.529 | 0.035 | 0.549 | -7.490 | 0.006 | -0.749 | 0.233 |
| WD | 7.154 | NC | 0.092 | 0.309 | 0.228 | 0.473 | 1.329 | 0.759 | -0.923 | 0.234 |
| Group1 | 0.787 | 123954.3 | 0.006 | 0.064 | 0.111 | 0.028 | -6.551 | 0.010 | -0.951 | 0.165 |
| Group2 | 7.855 | 1237082 | 0.001 | 0.898 | 0.005 | 0.927 | -24.69 | 0.000 | 2.846 | 0.994 |
| Group3 | 1.432 | NC | 0.009 | 0.314 | 0.032 | 0.255 | -20.66 | 0.000 | -1.709 | 0.023 |
| Total | 7.021 | 1105744.882 | 0.003 | 0.732 | 0.008 | 0.795 | -24.52 | 0.000 | -0.729 |  |

Note：ET, Expansion time; Group1, *P. rotundiloba*; Group2, *P. decomposita*; Group3, *P. rockii*; NC, not calculated

Table S6 Locations of populations used in Maxent

| species | Latitude | Longitude |
| --- | --- | --- |
| *Paeonia decomposita* | 30.079 | 102.087 |
| *Paeonia decomposita* | 30.237 | 102.184 |
| *Paeonia decomposita* | 30.425 | 102.278 |
| *Paeonia decomposita* | 30.533 | 102.027 |
| *Paeonia decomposita* | 30.894 | 101.928 |
| *Paeonia decomposita* | 31.084 | 101.864 |
| *Paeonia decomposita* | 31.321 | 102.016 |
| *Paeonia decomposita* | 31.706 | 102.023 |
| *Paeonia decomposita* | 31.892 | 101.028 |
| *Paeonia decomposita* | 31.914 | 102.102 |
| *Paeonia decomposita* | 31.999 | 102.019 |
| *Paeonia decomposita* | 31.288 | 102.476 |
| *Paeonia decomposita* | 31.027 | 102.254 |
| *Paeonia decomposita* | 32.084 | 103.157 |
| *Paeonia rotundiloba* | 31.592 | 103.46 |
| *Paeonia rotundiloba* | 31.498 | 103.189 |
| *Paeonia rotundiloba* | 32.341 | 103.702 |
| *Paeonia rotundiloba* | 34.013 | 103.389 |
| *Paeonia rotundiloba* | 31.917 | 103.405 |
| *Paeonia rotundiloba* | 31.936 | 103.688 |
| *Paeonia rotundiloba* | 31.591 | 103.7325 |
| *Paeonia rotundiloba* | 31.404 | 103.0639 |
| *Paeonia rockii* | 33.163 | 104.235 |
| *Paeonia rockii* | 32.882 | 104.717 |
| *Paeonia rockii* | 33.074 | 104.733 |
| *Paeonia rockii* | 32.615 | 106.136 |
| *Paeonia rockii* | 31.7193 | 111.1207 |
| *Paeonia rockii* | 33.0128 | 110.1675 |
| *Paeonia rockii* | 33.0164 | 104.4186 |
| *Paeonia rockii* | 33.5131 | 111.8537 |
| *Paeonia rockii* | 33.593 | 106.1272 |
| *Paeonia rockii* | 33.6696 | 106.2876 |
| *Paeonia rockii* | 33.6852 | 105.671 |
| *Paeonia rockii* | 33.9313 | 111.2064 |
| *Paeonia rockii* | 34.0543 | 105.7036 |
| *Paeonia rockii* | 34.0194 | 105.9131 |
| *Paeonia rockii* | 34.1333 | 112.0855 |
| *Paeonia rockii* | 34.1531 | 106.5183 |
| *Paeonia rockii* | 34.1572 | 107.8438 |
| *Paeonia rockii* | 34.5667 | 105.702 |
| *Paeonia rockii* | 34.6341 | 104.6736 |
| *Paeonia rockii* | 34.9105 | 108.9347 |
| *Paeonia rockii* | 35.0036 | 109.0508 |
| *Paeonia rockii* | 36.0026 | 108.6537 |
| *Paeonia rockii* | 36.5762 | 108.9504 |

**Figure Caption**

Fig.S1 Correlation between the number of groups (*K*) and genetic variance (*F*_CT_) in SAMOVA analysis

Fig.S2 Mismatch distribution analysis plots for each population based on nrITS data

Fig.S3 Mismatch distribution analysis plots for each population and SAMOVA-derived groups (6) 1–6 based on cpDNA data

Fig.S4 Bayesian skyline plot based on cpDNA sequences, showing the effective population size fluctuation throughout time

Fig.S5 Bayesian Skyline Plot based on nrITS sequences, showing the effective population size fluctuation throughout time

Fig. S6 Mismatch distribution analysis plots for all samples and SAMOVA-derived groups (3) 1-3 based on cpDNA data

Fig. S7 Mismatch distribution analysis plots for all samples and SAMOVA-derived groups 1-3 based on nrITS data

Fig.S1


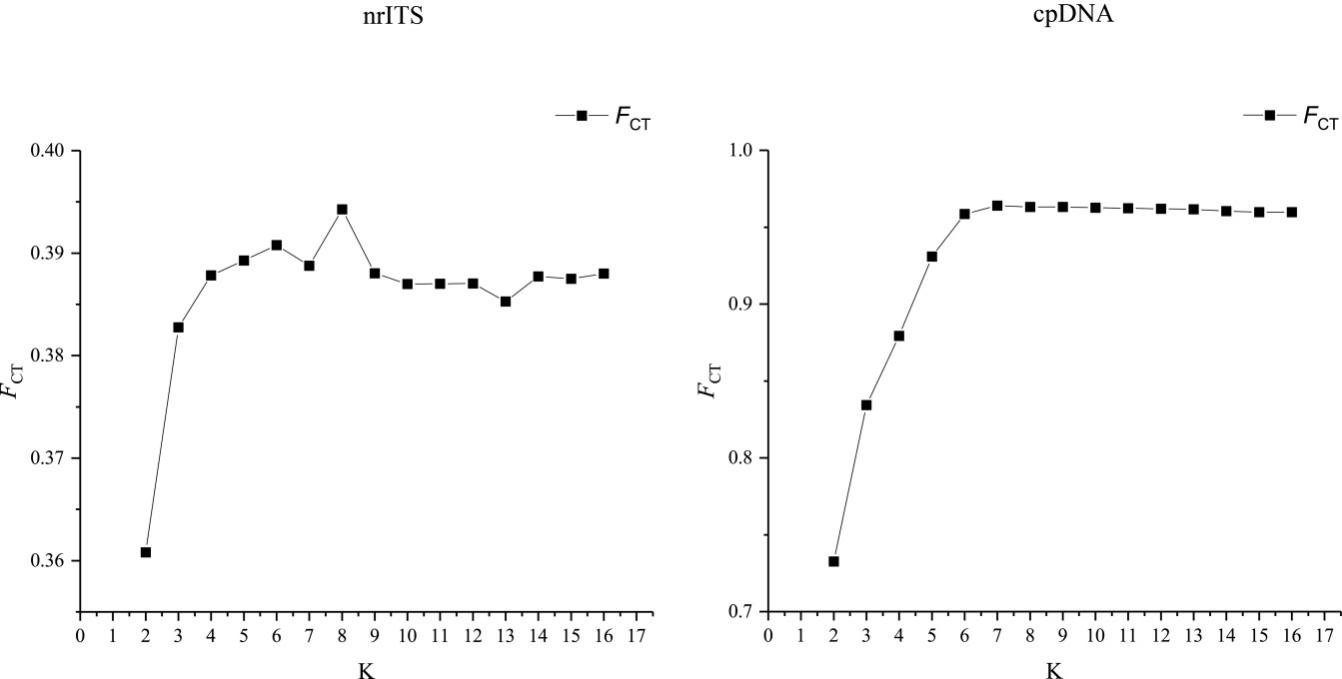


Fig.S2


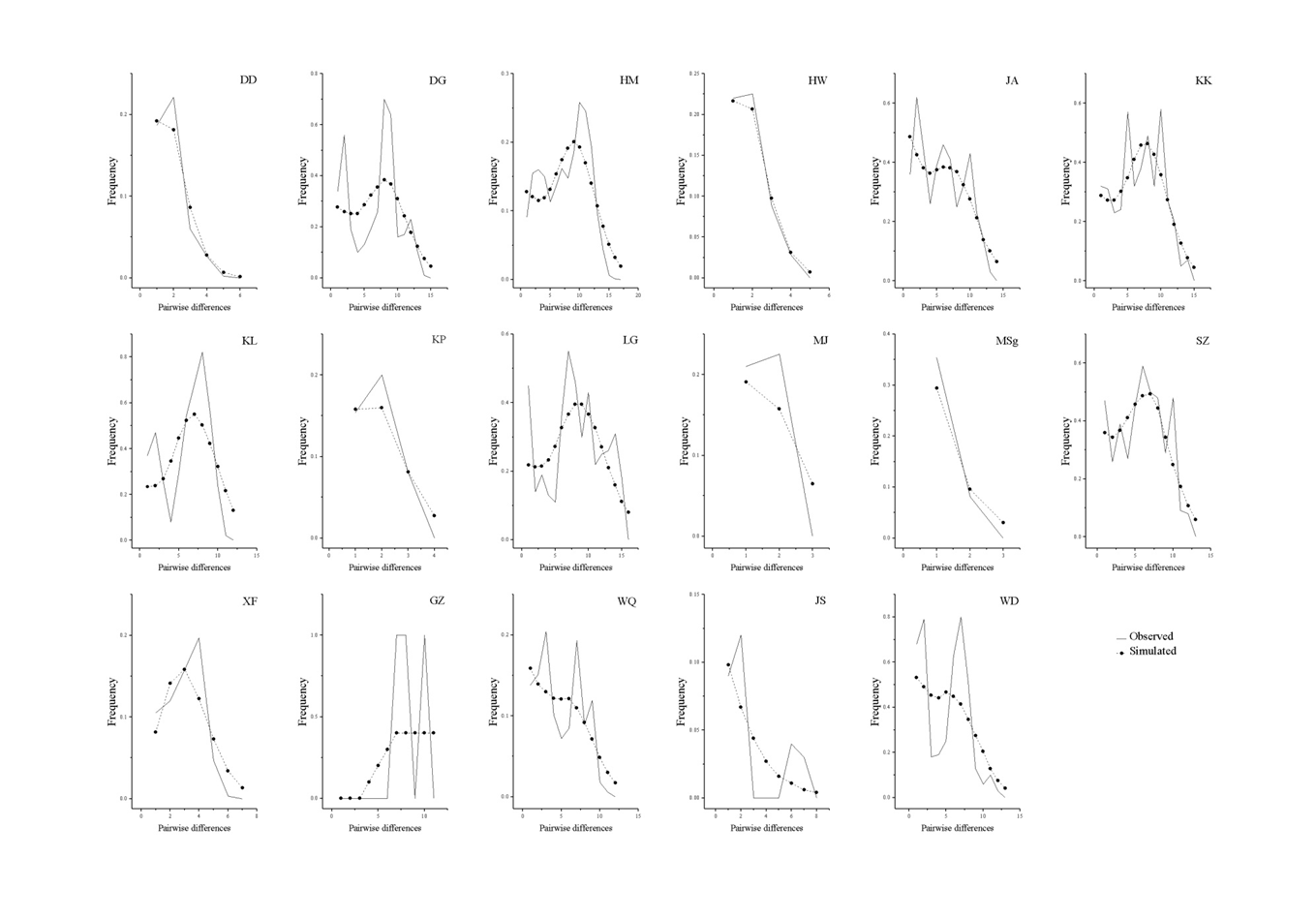


Fig.S3


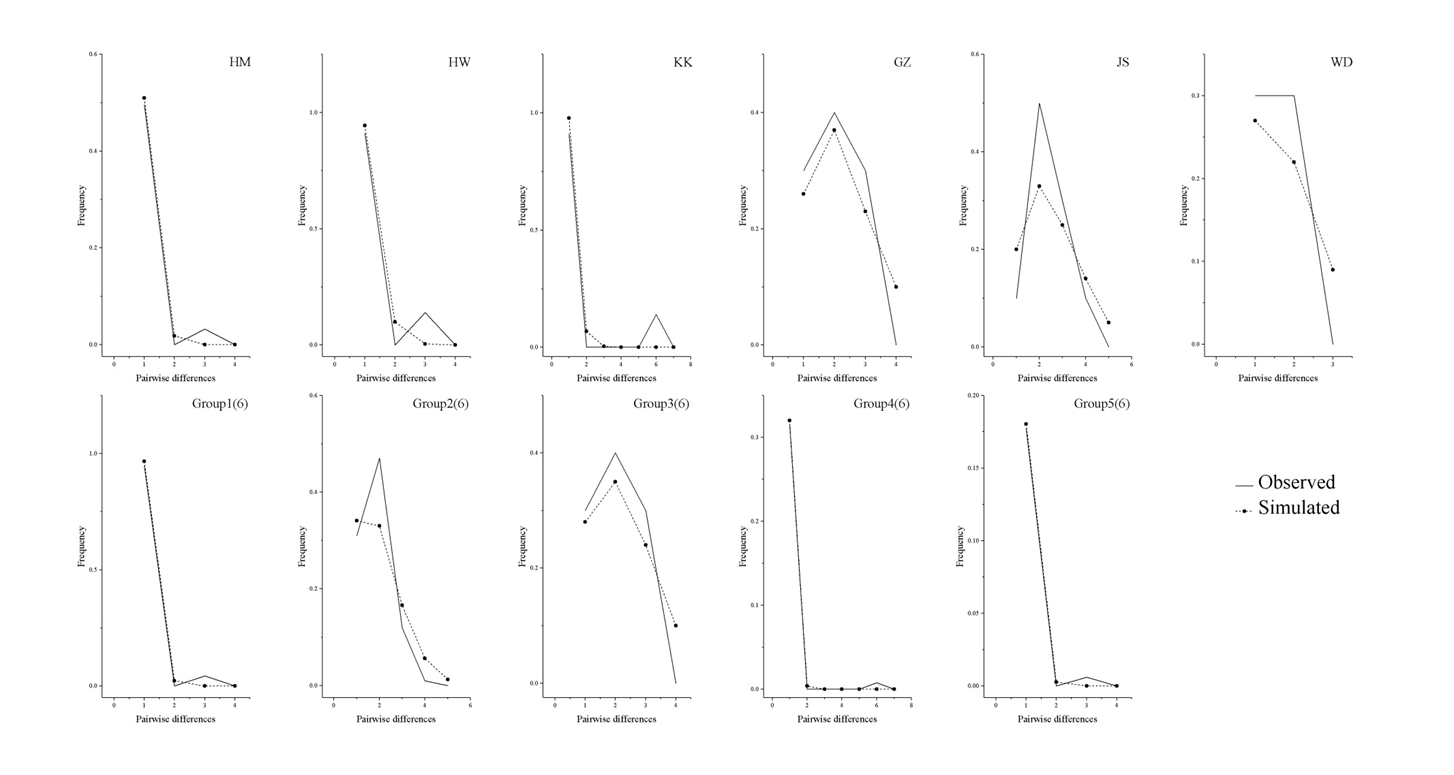


Fig.S4


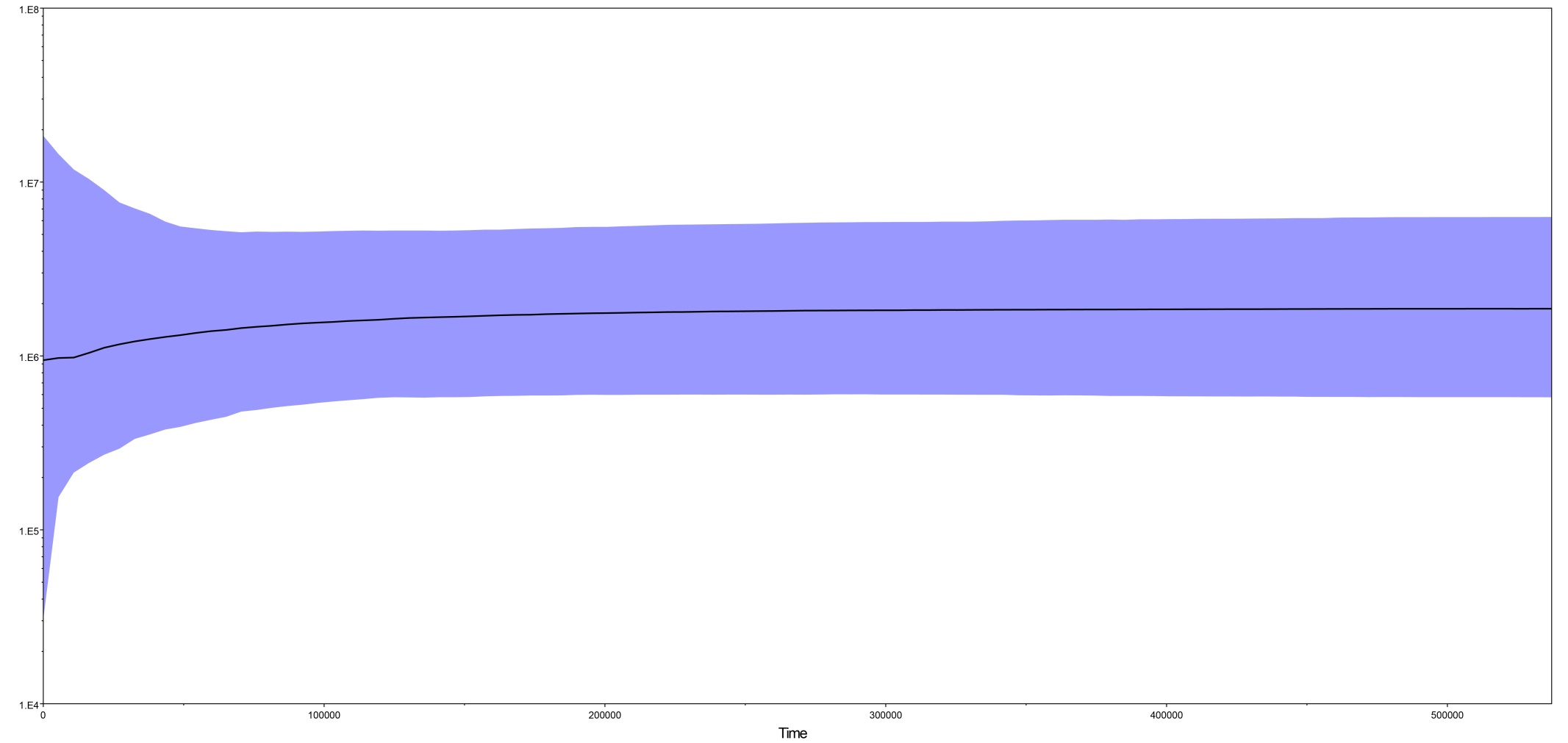


Fig.S5


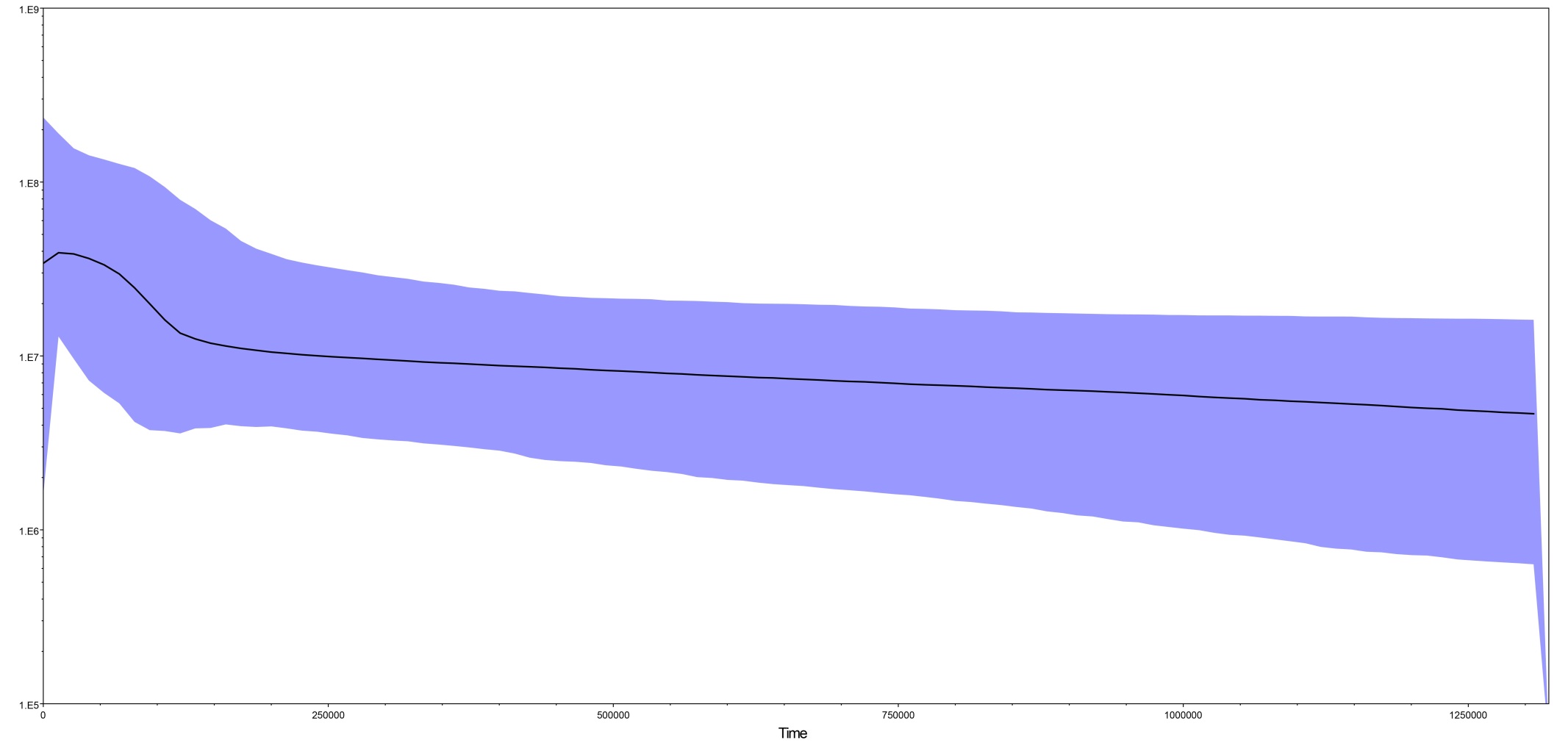


Fig. S6


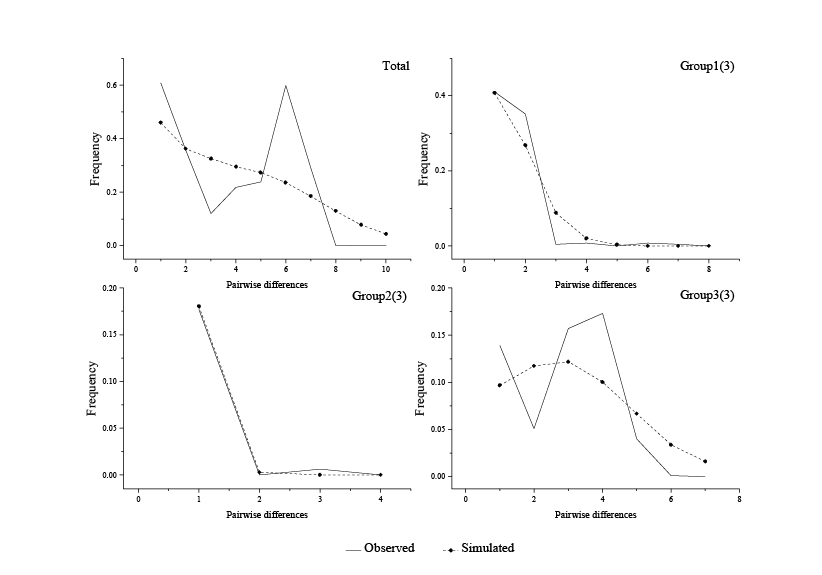


Fig. S7


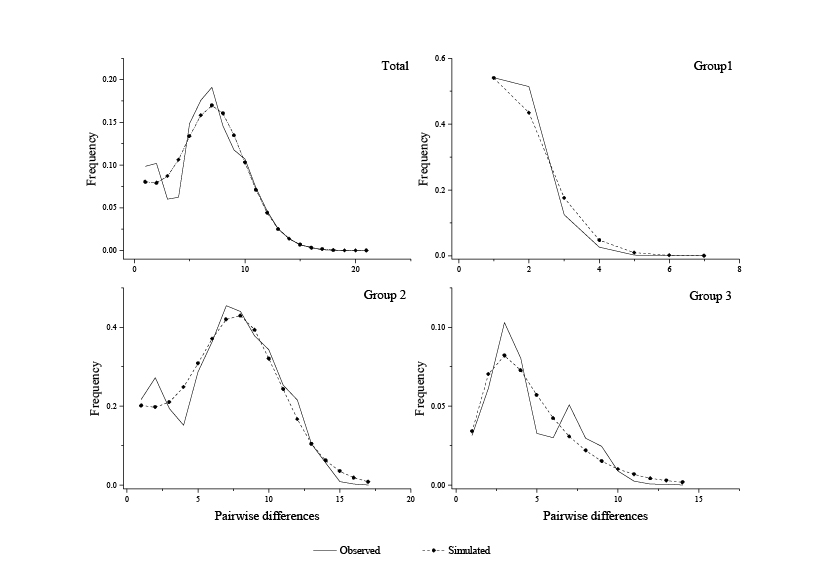

Supplement: Supplementary file 1 — Appendix S1: [file ECE3-13-e10073-s001.docx]
